# Supplementary material for: Engineering topological phases in triple HgTe/CdTe quantum wells
Source: Sci Rep. 2022 Feb 16;12:2617. doi: 10.1038/s41598-022-06431-0 (PMC8850558; doi:10.1038/s41598-022-06431-0)
Supplement: Supplementary file 1 — Supplementary Information. [file 41598_2022_6431_MOESM1_ESM.pdf]

Supplementary information for

# Engineering topological phases in triple HgTe/CdTe quantum wells

**G. J. Ferreira[1], D. R. Candido[2], F. G. G. Hernandez[3], G. M. Gusev[3], E. B. Olshanetsky[4], N. N. Mikhailov[4], and S. A. Dvoretsky[4]**

[1]Instituto de Física, Universidade Federal de Uberlândia, Uberlândia, MG 38400-902, Brazil
[2]Department of Physics and Astronomy, University of Iowa, Iowa City, Iowa 52242, USA
[3]Instituto de Física, Universidade de São Paulo, São Paulo, São Paulo 05508-090, Brazil
[4]Institute of Semiconductor Physics, Novosibirsk 630090, Russia

## $8 \times 8$ Kane model and material parameters

We use the Kane Hamiltonian[1–4] to obtain the subbands of the QWs described in this work. This Hamiltonian it is written in the basis $|\Gamma_6, \frac{1}{2}, \pm\frac{1}{2}\rangle$, $|\Gamma_8, \frac{3}{2}, \pm\frac{1}{2}\rangle$, $|\Gamma_8, \frac{3}{2}, \pm\frac{3}{2}\rangle$ and $|\Gamma_7, \frac{1}{2}, \pm\frac{1}{2}\rangle$ and reads as

$$\mathscr{H}^{8\times 8} = \begin{pmatrix} T & 0 & -\frac{1}{\sqrt{2}}Pk_+ & \sqrt{\frac{2}{3}}P\hat{k}_z & \frac{1}{\sqrt{6}}Pk_- & 0 & -\frac{1}{\sqrt{3}}P\hat{k}_z & -\frac{1}{\sqrt{3}}Pk_- \\ 0 & T & 0 & -\frac{1}{\sqrt{6}}Pk_+ & \sqrt{\frac{2}{3}}P\hat{k}_z & \frac{1}{\sqrt{2}}Pk_- & -\frac{1}{\sqrt{3}}Pk_+ & \frac{1}{\sqrt{3}}P\hat{k}_z \\ -\frac{1}{\sqrt{2}}Pk_- & 0 & U+V & -\bar{S}_- & R & 0 & \frac{1}{\sqrt{2}}\bar{S}_- & -\sqrt{2}R \\ \sqrt{\frac{2}{3}}P\hat{k}_z & -\frac{1}{\sqrt{6}}Pk_- & -\bar{S}_-^\dagger & U-V & C & R & \sqrt{2}V & -\sqrt{\frac{3}{2}}\tilde{S}_- \\ \frac{1}{\sqrt{6}}Pk_+ & \sqrt{\frac{2}{3}}P\hat{k}_z & R^\dagger & C^\dagger & U-V & \bar{S}_+^\dagger & -\sqrt{\frac{3}{2}}\tilde{S}_+ & -\sqrt{2}V \\ 0 & \frac{1}{\sqrt{2}}Pk_+ & 0 & R^\dagger & \bar{S}_+ & U+V & \sqrt{2}R^\dagger & \frac{1}{\sqrt{2}}\bar{S}_+ \\ -\frac{1}{\sqrt{3}}P\hat{k}_z & -\frac{1}{\sqrt{3}}Pk_- & \frac{1}{\sqrt{2}}\bar{S}_-^\dagger & \sqrt{2}V & -\sqrt{\frac{3}{2}}\tilde{S}_+^\dagger & \sqrt{2}R & U-\Delta & C \\ -\frac{1}{\sqrt{3}}Pk_+ & \frac{1}{\sqrt{3}}P\hat{k}_z & -\sqrt{2}R^\dagger & -\sqrt{\frac{3}{2}}\tilde{S}_-^\dagger & -\sqrt{2}V & \frac{1}{\sqrt{2}}\bar{S}_+^\dagger & C^\dagger & U-\Delta \end{pmatrix}, \tag{S.1}$$

with

$$T = E_c + \frac{\hbar^2}{2m_0}\left[(2F+1)k_\parallel^2 + \hat{k}_z(2F+1)\hat{k}_z\right] + a_c(2\varepsilon_{xx}+\varepsilon_{zz}), \tag{S.2}$$

$$U = E_v - \frac{\hbar^2}{2m_0}\left(\gamma_1 k_\parallel^2 + \hat{k}_z\gamma_1\hat{k}_z\right) + a_v(2\varepsilon_{xx}+\varepsilon_{zz}), \tag{S.3}$$

$$V = -\frac{\hbar^2}{2m_0}(\gamma_2 k_\parallel^2 - 2\hat{k}_z\gamma_2\hat{k}_z) + b(\varepsilon_{xx}-\varepsilon_{zz}), \tag{S.4}$$

$$R = -\frac{\hbar^2}{2m_0}\sqrt{3}(\mu k_+^2 - \bar{\gamma}k_-^2), \tag{S.5}$$

$$\bar{S}_\pm = -\frac{\hbar^2}{2m_0}\sqrt{3}k_\pm\left(\{\gamma_3,\hat{k}_z\} + [\kappa,\hat{k}_z]\right), \tag{S.6}$$

$$\tilde{S}_\pm = -\frac{\hbar^2}{2m_0}\sqrt{3}k_\pm\left(\{\gamma_3,\hat{k}_z\} - \frac{1}{3}[\kappa,\hat{k}_z]\right), \tag{S.7}$$

$$C = \frac{\hbar^2}{m_0}k_-[\kappa,\hat{k}_z]. \tag{S.8}$$

Here, all the parameters depend on the coordinate $z$, which is set along the QW growth direction. $E_c$ and $E_v$ correspond to the energies at $k_x = k_y = 0$ of the $|\Gamma_6\rangle$ and $|\Gamma_8\rangle$ bands, respectively, and $\Delta$ quantifies the split-off energy separation, i.e., the energy between $|\Gamma_8\rangle$ and $|\Gamma_7\rangle$ bands. Here, $[A,B] = AB - BA$ denotes the commutator between the $A$ and $B$ operators while $\{A,B\} = AB + BA$ denotes their anti-commutator. $P$ is the Kane parameter, $m_0$ is the free electron mass, $\gamma_i$ are the Luttinger parameters[5], which together with $\kappa$ and $F$ accounts for the effective mass correction due to the remote bands.

## Effective 2D model coefficients

The coefficients for the effective 2D model[6,7] are calculated from the $\boldsymbol{k}\cdot\boldsymbol{p}$ method in the basis of *single QW eigenstates* from the $(k_x,k_y)=0$ numerical solutions of the $8\times 8$ Kane model, which we express here as

$$\mathscr{H}^{8\times 8} = H_0 + H_x k_x + H_y k_y + H_{xx} k_x^2 + H_{yy} k_y^2 + H_{xy} k_x k_y, \tag{S.9}$$

such that the indexes $\mu$ in each $H_\mu$ indicate their corresponding $k_x$ and $k_y$ powers, while their $z$ and $k_z = -i\partial_z$ dependence is implied within each term. This notation is useful to express the matrix elements in the following discussion by explicitly showing the $(k_x,k_y)$ terms, while omitting the $z$ directions that is integrated on each matrix element.

Following the Löwdin partitioning, we calculate the effective model defining a set $A$ of eigenstates from the E1 and H1 solutions for the QW $\nu$, which we label as $A = \{|H_{1\pm}^\nu\rangle, |E_{1\pm}^\nu\rangle\}$, and a set $B$ of *remote bands* as all remaining eigenstates $|b\rangle$. In practice, the sum over the remote bands $b\in B$ is truncated up to $\sim 20$ eigenstates above and below the Fermi energy, and its convergence is verified. Additionally, we emphasize that the basis sets A and B are extracted from single QW models of $\mathscr{H}^{8\times 8}$, while, in the matrix elements below, $\mathscr{H}^{8\times 8}$ is set as the triple QW. Therefore, the basis states are only approximately eigenstates of $H_0$. The resulting model was shown in the main text, and up to second order in Löwdin's perturbation, the matrix elements of the effective Hamiltonian for $a\in A$ are

$$C_\nu + M_\nu \approx \langle E_{1\pm}^\nu | H_0 | E_{1\pm}^\nu\rangle + \delta C_\nu + \delta M_\nu, \tag{S.10}$$

$$C_\nu - M_\nu \approx \langle H_{1\pm}^\nu | H_0 | H_{1\pm}^\nu\rangle + \delta C_\nu - \delta M_\nu, \tag{S.11}$$

$$A_\nu \approx \langle E_{1\pm}^\nu | H_x | H_{1\pm}^\nu\rangle, \tag{S.12}$$

$$D_\nu + B_\nu \approx \langle E_{1\pm}^\nu | H_{xx} | E_{1\pm}^\nu\rangle + \sum_{b\in B} \frac{\langle E_{1\pm}^\nu | H_x |b\rangle\langle b| H_x | E_{1\pm}^\nu\rangle}{\varepsilon^0_{E_{1\pm}^\nu} - \varepsilon^0_b}, \tag{S.13}$$

$$D_\nu - B_\nu \approx \langle H_{1\pm}^\nu | H_{xx} | H_{1\pm}^\nu\rangle + \sum_{b\in B} \frac{\langle H_{1\pm}^\nu | H_x |b\rangle\langle b| H_x | H_{1\pm}^\nu\rangle}{\varepsilon^0_{H_{1\pm}^\nu} - \varepsilon^0_b}. \tag{S.14}$$

$$\delta C_\nu + \delta M_\nu \approx \sum_{b\in B} \frac{|\langle E_{1\pm}^\nu | H_0 |b\rangle|^2}{\varepsilon^0_{E_{1\pm}^\nu} - \varepsilon^0_b} + \cdots, \tag{S.15}$$

$$\delta C_\nu - \delta M_\nu \approx \sum_{b\in B} \frac{|\langle H_{1\pm}^\nu | H_0 |b\rangle|^2}{\varepsilon^0_{H_{1\pm}^\nu} - \varepsilon^0_b} + \cdots. \tag{S.16}$$

Above, the $\delta C_\nu$ and $\delta M_\nu$ terms are higher order corrections that arise because the single QW basis states are not exact eigenstates of the triple QW $H_0$. In practice, these corrections converge slowly and we consider $\delta C_\nu$ and $\delta M_\nu$ as free parameters to adjust the band edges. For the results presented in the main text, we have neglected these corrections in Fig. 3, since it does not affect the results qualitatively. In contrast, in Fig. 5(a)-(b), near the phase transition, we have used these parameters to adjust the band edges, since it is critical to recover the correct phase.

**Table S.1.** Parameters used in the $8\times 8$ Kane Hamiltonian for HgTe/$Hg_{1-x}Cd_xTe$ heterostructures. For a concentration $x$ the alloy gap is $E_g(x) = (1-x)E_g^{\mathrm{HgTe}} + xE_g^{\mathrm{CdTe}} - 0.132x(1-x)$, while all other parameters follow a linear interpolation with $x$. Additionally, we have $P^2 = \hbar^2 E_p/2m_0$, $E_c = E_g + E_v$, $\mu = (\gamma_3-\gamma_2)/2$, $\bar\gamma = (\gamma_3+\gamma_2)/2$, $\varepsilon_{xx} = [a^{\mathrm{CdTe}} - a(x)]/a(x)$, and $\varepsilon_{zz} = -2C_{12}\varepsilon_{xx}/C_{11}$.

| Parameter | HgTe | CdTe | Parameter | HgTe | CdTe |
|---|---|---|---|---|---|
| $E_p$ [eV] | 18.8 | 18.8 | $a$ [Å] | 6.46 | 6.48 |
| $E_g$ [eV] | -0.303 | 1.606 | $a_c$ [eV] | -2.380 | -2.925 |
| $E_v$ [eV] | 0 | -0.570 | $a_v$ [eV] | 1.31 | 0 |
| $\Delta$ [eV] | 1.08 | 0.91 | $b$ [eV] | -1.5 | -1.2 |
| $F$ | 0 | -0.09 | $C_{11}$ [GPa] | 53.6 | 53.6 |
| $\gamma_1$ | 4.1 | 1.47 | $C_{12}$ [GPa] | 36.6 | 37 |
| $\gamma_2$ | 0.5 | -0.28 | | | |
| $\gamma_3$ | 1.3 | 0.03 | | | |
| $\kappa$ | -0.4 | -1.31 | | | |

For the inter-well couplings $v_\mu(\boldsymbol{k})$ the coefficients depend upon the eigenstates from neighboring QWs, which we label as $\nu$ and $\nu'$ next. For instance, for $\mu = LC$ we consider single QW eigenstates from $\nu = L$ and $\nu' = C$. Thus, generically, these coefficients read as

$$\Delta_{E1,\mu} = 2\left\langle E^{\nu}_{1\pm}\right| H_0 \left| E^{\nu'}_{1\pm}\right\rangle, \tag{S.17}$$

$$\Delta_{H1,\mu} = 2\left\langle H^{\nu}_{1\pm}\right| H_0 \left| H^{\nu'}_{1\pm}\right\rangle, \tag{S.18}$$

$$\alpha_{\mu} = 2\left\langle E^{\nu}_{1\pm}\right| H_x \left| H^{\nu'}_{1\pm}\right\rangle, \tag{S.19}$$

with $\Delta_{E1,\mu} = \Delta_{0,\mu} + \Delta_{z,\mu}$, and $\Delta_{H1,\mu} = \Delta_{0,\mu} - \Delta_{z,\mu}$.

## Semi-metallic phase

In the main text, Fig. 1 shows the band structure for $t = 3$ nm and varying $d_0 = \{5, 6, 7, 8.5\}$ nm. Complementary, here, Figure S.1 shows the band structure in the semi-metallic regime for $t = 3$ nm and $d_0 = 12$ nm.

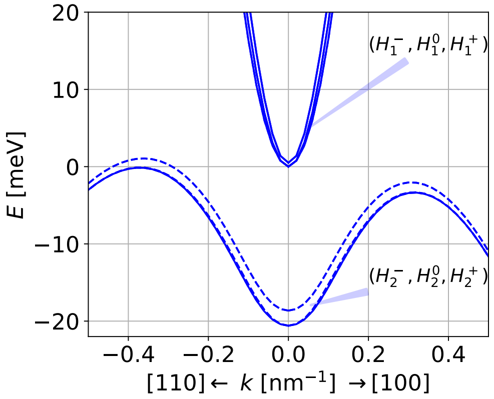


**Figure S.1.** Band structure calculated with the $8 \times 8$ Kane model for barrier width $t = 3$ nm and large quantum well width $d_0 = 12$ nm. As can be seen from Fig. 2(a) from the main text, for large $d_0$ the E subbands cross the second set of H subbands and the figure shows only the H subbands near the Fermi level $E_F = 0$ meV. In this case the indirect gap is closed and the system is in the semi-metallic regime.

## References


1. Bir, G. L. & Pikus, G. E. *Symmetry and strain-induced effects in semiconductors*, vol. 484 (Wiley New York, 1974).
2. Bastard, G. *Wave Mechanics Applied to Semiconductor Heterostructures* (Les Editons de Physique, Paris, 1988).
3. Voon, L. C. L. Y. & Willatzen, M. *The kp method: electronic properties of semiconductors* (Springer Science & Business Media, 2009).
4. Winkler, R. *Spin-orbit coupling effects in two-dimensional electron and hole systems*, vol. 191 of *Springer Tracts in Modern Physics* (Springer-Verlag, Berlin, Heidelberg, 2003).
5. Luttinger, J. M. & Kohn, W. Motion of Electrons and Holes in Perturbed Periodic Fields. *Phys. Rev.* **97**, 869–883, DOI: 10.1103/PhysRev.97.869 (1955).
6. Michetti, P., Budich, J. C., Novik, E. G. & Recher, P. Tunable quantum spin Hall effect in double quantum wells. *Phys. Rev. B* **85**, 125309, DOI: 10.1103/physrevb.85.125309 (2012).
7. Michetti, P. & Trauzettel, B. Devices with electrically tunable topological insulating phases. *Appl. Phys. Lett.* **102**, 063503, DOI: 10.1063/1.4792275 (2013).
